# Effects of a single session low-threshold digital intervention for procrastination behaviors among university students (Focus): Findings from a randomized controlled trial

**DOI:** 10.1016/j.invent.2024.100741

**Published:** 2024-04-08

**Authors:** Katarina Åsberg, Marie Löf, Marcus Bendtsen

**Affiliations:** a Department of Health, Medicine and Caring Sciences, Linköping University, Sweden; b Department of Biosciences and Nutrition, Karolinska Institute, Sweden

**Keywords:** Procrastination, Telemedicine, University students, Mental health, Public health

## Abstract

**Introduction:**

Procrastination behaviors are common among university students, and have been found to be associated with stress, symptoms of depression, anxiety, and poorer academic performance. There is a need for interventions that can reach students at scale, and therefore this study aimed to estimate the effects of a single session low-threshold digital intervention (Focus) for procrastination behaviors among university students in Sweden.

**Methods and analysis:**

A two-arm, parallel groups (1:1), single blind randomized controlled trial was conducted between February 8 to April 26, 2023. The study used email to invite university students across Sweden to participate in the trial. Both the intervention and the control group were invited to assess their current procrastination behaviors using the Pure Procrastination Scale (PPS). The intervention group immediately received feedback and behavior change advice by means of an interactive website, while the control group was shown their total PPS score without any further feedback. Students were included in the study if they scored 20 points or more on the PPS. Our primary outcome was procrastination behavior measured at 2 months post-randomization. Analyses were conducted using multilevel regression models estimated with Bayesian inference.

**Results:**

A total of 2209 participants (intervention: 1109, control: 1100) were randomized. The average age of participants was 26.4 years (SD = 7.8) and 65 % were women (*n* = 1442). The mean PPS score at baseline was 35.6 points (of a maximum of 60). Primary outcome data were available for 45 % (*n* = 498) of the intervention group and 55 % (*n* = 601) of the control group. The evidence suggested no marked difference between groups regarding any of the outcomes, although there was weak evidence of lower physical activity in the intervention group. Qualitative findings from open-ended responses uncovered a variety of views on procrastination and perceived problems that may follow. Those not feeling supported by Focus explained having troubles adopting the advice given and converting their intentions into action without more continuous support.

**Conclusions:**

Access to a single session of feedback and behavior change advice by means of an interactive website did not produce differential self-reported procrastination among university students who took the opportunity to self-assess their behaviors. The findings are limited by assessment reactivity due to screening at baseline and attrition to follow-up.

## Highlights

• Procrastination negatively impacts students' mental health and academic performance.
• There is a need for interventions that have an increased reach among students.
• A single session digital intervention did not produce lower self-reported procrastination.
• User evaluation revealed that most students felt no need for support.
• Students expressed difficulties adopting advice without continuous support.

## 1 Introduction

Procrastination is often described as a self-regulatory failure (Fernie et al., 2017) and is defined as the irrational and voluntary delay of an intended course of action despite expecting to be worse off for the delay (Steel, 2007). Procrastination has a negative impact on mental health and is associated with stress, symptoms of depression, and anxiety (Johansson et al., 2023; Beutel et al., 2016). It seems that procrastination behaviors are common among university students and previous studies indicate that approximately 50 % of students are likely to procrastinate their academic assignments or tasks (Atalayin et al., 2018; Ferrari et al., 2009). Academic procrastination can be seen as a within-person risk factor for academic difficulties, and procrastination has been shown to be negatively correlated with academic performance (Kim and Seo, 2015; Kljajic and Gaudreau, 2018). In addition, procrastination also appears to have a negative effect on lifestyle behaviors including delaying and avoiding health-promoting behaviors (Sirois and Giguère, 2018; Sirois et al., 2023). Interview studies which have focused on university students' experiences of health and health-related behaviors during the academic years, has highlighted that procrastination and study-related stress out-compete healthy behaviors such as physical activity, sleep hygiene, and healthy cooking (Åsberg et al., 2022).

Previous research evaluating the effectiveness of cognitive behavior therapy (CBT) interventions for procrastination has shown promise. These programs are delivered both in face-to-face and digital settings (iCBT), and both with and without additional support from a therapist (Rozental et al., 2015a; Rozental et al., 2018; Eckert et al., 2018; Gagnon et al., 2019; Lukas and Berking, 2018; Mutter et al., 2023). Rozental et al. (Rozental et al., 2015a) evaluated guided and unguided self-help using iCBT in the general population, showing moderate effects on procrastination. In another study, they compared iCBT with group therapy among university students with severe procrastination symptoms, but found no statistically significant differences between groups after treatment (Rozental et al., 2018). More recently, Mutter et al. (Mutter et al., 2023) conducted a non-inferiority trial of a digital intervention guided by either an avatar providing immediate standardized feedback, or by a human psychologist providing semi-standardized feedback digitally, with the evidence suggesting that the avatar coach was not inferior to human guidance. Finally, Eckert et al. (Eckert et al., 2018) evaluated a two-week unguided online intervention for university students to overcome procrastination, with and without additional text messages, which showed small to medium effects on procrastination. However, previous research shows that engaging in iCBT treatment programs can be demanding, where too many commitments can make it hard for students to stay motivated and stick with their treatment schedule over time (Rozental et al., 2015b).

Since procrastination has a negative impact on student's mental health and academic performance, we conducted a study of a single session low-threshold digital intervention for procrastination behaviors called *Focus.* The primary aim of the study was to estimate the effects of Focus on procrastination behaviors, measured using the 12-item Pure Procrastination Scale (PPS), among university students in Sweden. A secondary aim was to explore students' perceptions of Focus, with an emphasis on their needs, perceived usefulness, and acceptability of the intervention. The Focus intervention is a digital intervention based on a public health approach (Rose, 1981) of targeting the whole population rather than high-risk groups, and is intended to be proactive and invite students to self-assess their level of procrastination. As such, Focus is an adjunct to psychological treatment and is intended to help students identify procrastination as a problem, and if appropriate seek more help if required.

## 2 Methods

This study was a single blind, two-arm parallel groups (1:1), randomized controlled trial (RCT). The trial was registered prospectively (on February 03, 2023) in the ISRCTN registry (ISRCTN13533793) and received ethical approval from the Swedish Ethical Review Authority on August 24, 2022 (Dnr 2022-00353). A SPIRIT study protocol (Chan et al., 2013) including a full description of the intervention together with a statistical analysis plan was submitted prior to enrolment (Åsberg and Bendtsen, 2023). There were no deviations from the study protocol. This report follows the guidelines of the CONSORT statement (Schulz et al., 2010).

### 2.1 Participants, recruitment, randomization, and blinding

The study was undertaken simultaneously at four universities in Sweden (Halmstad University, Linköping University, Luleå University of Technology, and Mälardalen University). Students at the participating universities, including both program and individual course students, were sent email invitations by representatives of each university's student health care center. The invitation asked students if they would consider participating in a study in which they would be asked to assess their procrastination behaviors.

The email invitation contained a hyperlink to a webpage presenting study information and informed consent materials. Students who consented were asked to respond to a web-based questionnaire which collected baseline data and checked for eligibility (please see Appendix A for all questions asked at baseline and follow-up). Eligible participants were students who completed the baseline questionnaire and scored 20 points or more on the Pure Procrastination Scale (PPS). Email invitations and study materials were only available in Swedish, excluding those not understanding Swedish enough to participate. Participants did not receive financial compensation for participating.

After completing the baseline questionnaire, participants were randomized to one of two groups: the Focus intervention (intervention group) or a non-treatment condition (control group). Randomization was done using block randomization (with random block sizes of 2 and 4). All study procedures, from recruitment, informed consent, eligibility screening, randomization, baseline assessment, intervention delivery, and follow-ups were automated and computerized. Neither research personnel nor participants were able to discover or manipulate the randomization sequence. Regarding blinding, due to the study's automation and digital delivery, research personnel were blinded both before and after allocation. Meaning they were not directly involved in participant recruitment, eligibility screening, randomization, or allocation, nor were they involved in assessing study outcomes.

### 2.2 Interventions

Following baseline assessment, participants allocated to the control group were shown their total PPS score (summary score), with the minimum and maximum on the scale presented, with a recommendation to read more about procrastination at their local student health care center's website.

Participants allocated to the intervention group were after baseline taken to a website that showed the Focus intervention. This single-session intervention which consisted of graphical and written content took participants approximately 5–10 min to complete depending on how much participants interacted with the content. Graphical and written feedback was shown based on participants current procrastination behaviors (assessed by PPS) together with behavior change advice. The graphical feedback utilized a traffic-light symbolism employing the colors green, yellow, orange, and red to illustrate different ranges of scores on PPS, as an intuitive feedback on participants total score. The written feedback contained general information about goal setting and planning and provided participants with the opportunity to set a goal and create a plan for the following week. The feedback participants received also included tips on how to change procrastination behaviors which were tailored based on the constructs outlined in Svartdal & Steels 3-factor model: decisional, implemental, timeliness and promptness delay (Svartdal and Steel, 2017). Decisional procrastination, assessed by question 1–3, involves delaying decisions or avoiding making choices. Implemental procrastination, assessed by question 4–8, refers to postponing task execution even after a decision has been made. Meanwhile, timeliness and promptness delay relate to effective time management and acting promptly when necessary, such as meeting deadlines or allocating time efficiently. Participants received tailored tips based on their highest scoring construct, addressing the aspects of procrastination where they faced the most pronounced tendencies. For instance, those scoring high on decisional procrastination received advice related to decision-making. For example *“Take control- Do you have all the information you need to be able to make a decision, or do you need to check something or get an answer from someone? Clear up any ambiguities to make decisions easier”.* Or for implemental procrastination participants received tips like *“Try a pomodoro-timer- Use the Pomodoro method and work in focused study sessions of, for example 30, 45, or 60 minutes of work with a subsequent 5-10 minute break. Remember that four productive units makes a good study day”.*

All intervention content aimed to enhance self-reflection and stimulation of self-reinforcement, in line with self-regulation theory (Kanfer and Gaelick-Buys, 1991). Lastly, Focus was also inspired from existing evidence-based research on procrastination treatment programs using internet-based cognitive behavioral therapy (iCBT) (Rozental et al., 2015a; Rozental et al., 2018). The content and usability testing of the intervention was designed and developed in iterative steps, in dialogue and collaboration with seven professionals at the student health care centers in Sweden. In 2022, a series of multidisciplinary group meetings were held where procrastination behaviors among university students were discussed, problematized, and defined in behavioral terms. Intervention content and recruitment strategies were developed based on these meetings.

### 2.3 Outcomes

#### 2.3.1 Primary and secondary outcome measures

The primary outcome was procrastination behavior at 2 months post-randomization. Procrastination was assessed by the 12-item PPS, with each item on the scale scored on a five-point Likert scale (12–60). PPS was originally developed by Steel (Steel, 2010) and translated to Swedish by Rozental et al. (Rozental et al., 2014). PPS is proven to provide a comprehensive measure of procrastination, and the scale is composed of 12 items selected from three established procrastination scales focusing on different aspects: the Decisional Procrastination Scale (DPS), which primarily measures decisional delay, the Adult Inventory of Procrastination Scale (AIP), where most items address timeliness and lateness, and the General Procrastination Scale (GPS), which focuses on implemental delay. (Svartdal and Steel, 2017). The scale has not been validated in the context of this study; however, use of the scale is supported in non-clinical settings (Rozental et al., 2014), and higher scores have previously been used to indicate greater difficulties with procrastination. Reliability of PPS within the study sample appeared high at baseline (Cronbach's alpha = 0.91) and follow-up (Cronbach's alpha = 0.91).

Secondary outcomes were anxiety symptoms, stress symptoms, and lifestyle behaviors. Anxiety was measured using the validated seven item Generalized Anxiety Disorder scale (GAD-7), translated to Swedish by Pfizer (Pfizer Inc, n.d.), and demonstrated high reliability in this sample (Cronbach's alpha = 0.90) (Spitzer et al., 2006). The scale is frequently used to screen for symptoms of anxiety and has, for instance, been used in research including the general population of Sweden (Johansson et al., 2013). Experienced levels of stress was measured by using the four item short form perceived stress scale (PSS-4) (Cohen et al., 1983; Vallejo et al., 2018). The original 14-item version (PSS-14) has been validated and translated to Swedish by Eskin and Par (Eskin and Parr, 1996), and the shorter 10-item version (PSS-10) by Nordin (Nordin and Nordin, 2013). Although not recommended as a measure of perceived stress due to issues with multidimensionality and reliability (Rozental et al., 2023) we decided to include the short version to reduce participant burden while still being able to measure some indicator of perceived stress. The reliability of PSS-4 was barely acceptable in our sample (Cronbach's alpha = 0.71), emphasizing that it may not be appropriate as a primary outcome in a study of stress. To explore whether procrastination behaviors were barriers for healthy lifestyle behaviors, we included measures of alcohol, diet, physical activity, and smoking. Total weekly alcohol consumption was measured by asking participants the number of standard drinks consumed in the past week, and frequency of heavy episodic drinking was assessed by asking participants how many times they consumed 4 or more standard drinks of alcohol on one occasion the past month. In Sweden, a standard drink of alcohol is defined as 12 g of pure alcohol, for example 125 ml wine or half a pint of beer. Both outcomes for alcohol are part of the core outcome set for brief alcohol interventions (Shorter et al., 2021). Consumption of candy, snacks, and sugary drinks was measured using a questionnaire designed by the Swedish National Board of Health and Welfare, and further modified to also include portion sizes (Socialstyrelsen, 2018). Candy and snacks were measured using a single question regarding number of servings consumed last week, and sugary drinks consumption were measured by a question regarding the number of units (33 cl) of sugary drinks consumed the past week. Moderate and vigorous physical activity were estimated by summing responses to two questions regarding the number of minutes spent on moderate and vigorous physical activity, respectively, during the past week.

Finally, participants allocated to the intervention group were, at the 2-month follow-up, asked four questions about perceived acceptability and usefulness of the intervention. Intervention group participants were also given the option of leaving a comment describing their needs and how Focus did or did not support them.

#### 2.3.2 Follow-up procedures

Follow-up 2 months post-randomization was initiated by sending an email to all randomized participants including a hyperlink to a web-based questionnaire. A total of two email-reminders were sent one week apart to those not responding. Students had one week to complete the follow-up assessments after receiving the email-reminder.

### 2.4 Sample size

Bayesian analysis (Bendtsen, 2018) was used to estimate the effects of the intervention and we used a Monte Carlo study to determine the number of required participants to achieve a desired precision of estimates. We assumed a standardized effect of 0.35 Cohen's d of the intervention on the primary outcome. The simulations suggested that data from 1000 participants were required for analysis under the requirement that at least 80 % of the time the posterior probability of effect was at least 95 %. We assumed a 50 % attrition rate, meaning that 2000 randomized participants were required.

Since the Focus intervention was novel, there were no previous studies to rely on regarding the determination of effect sizes. However, a previous study that compared guided and unguided self-help, using internet-based cognitive behavior therapy with a wait-list control, observed effects sizes ranging from 0.5 to 0.7 Cohen's d (Rozental et al., 2015a). We anticipated that our single session intervention would be less effective than a full therapy program, and therefore decided that the smallest effect size that we did not want to miss was a Cohen's d of 0.35.

### 2.5 Statistical methods

All participants were analyzed in the groups to which they were randomized (intention-to-treat). All models included adaptive intercepts for each university (multilevel). Missing data was handled using multiple imputation with chained equations (with 200 imputed data sets using 30 iterations). Both available data and imputed analyses were used to interpret findings.

The primary outcome (PPS) was standardized and analyzed using linear regression, adjusted for age, sex, and PPS at baseline. The secondary outcome measures anxiety, stress, weekly consumption of candy and snacks, and physical activity were modelled equivalently as PPS. Frequency of heavy episodic drinking, weekly alcohol consumption, and weekly consumption of sugary drinks were analyzed using negative binomial regression, adjusted for age, sex and PPS at baseline.

Effect modification analyses were conducted by estimating regression models with interactions between treatment allocation and age, sex, and baseline PPS. Models were compared using the Widely Applicable Information Criterion (WAIC) to decide if interaction models were informative in comparison to non-interaction models. Regarding attrition analyses, logistic regression was used to estimate the odds ratio of not responding to follow-up with respect to age, sex, and baseline PPS.

All models were estimated using Bayesian inference (Bendtsen, 2018). In the Bayesian paradigm, the emphasis is on estimating a distribution of effect estimates conditional on the data and one's prior beliefs. This distribution is referred to as the *posterior* distribution (i.e., after the data). A consequence of doing so is that one does not need to dichotomize evidence into the existence or non-existence of an effect, but rather, the entire distribution can be studied and used for scientific inference. This allows us to directly reason about the probability of different effect sizes. It is illustrative to report the *probability of effect* as the probability that the effect estimate is greater (or less) than the null value. For instance, in the case of this study, the probability that the mean difference in PPS scores between groups is less than 0. In contrast, traditional null-hypothesis testing assumes that the effect is exactly the null and then calculates the likelihood of the collected data under this assumption. If the data is unlikely under the null assumption (according to a predefined threshold), then the assumption is rejected and instead one declares that the effect estimate is not the null, and thus, there “exists” an effect. On the other hand, if the assumption cannot be rejected, one is left with ambivalence regarding the nature of the effect. This is where the difference in paradigms is perhaps most tangible because the product of a Bayesian analysis is always a distribution over effect sizes which can be used to reason about the effect of an intervention without an initial assertion of the existence of the effect or not. In other words, in the Bayesian paradigm, there may or may not exists a “true” population effect, however, we only concern ourselves with what the data from the current study tells us about the estimated effect.

In this study, we will report the posterior probability of effect, i.e., the probability that there is a difference between groups with respect to the outcome measures. We will also use medians of posterior distributions as point estimates of effect estimates with 95 % compatibility intervals defined as the 2.5 and 97.5 percentiles of the posterior distributions. To encode a conservative prior belief about the effects of Focus, we used Student's t priors for covariate coefficients in our regression models, and normal priors were used for adaptive intercepts with half-Student's t hyper-priors for the standard deviation, and half-Student's t priors for main standard deviations and dispersion parameters. All Student's t priors had 3 degrees of freedom and a scale of 2.5.

#### 2.5.1 Qualitative analysis

We conducted an inductive content analysis (Elo and Kyngäs, 2008) of the intervention group participants' open-ended responses describing their needs and how the intervention did or did not support them. First, all responses were read by authors KÅ and MB to grasp the data as a whole. Second, responses were exported into NVivo (version 1.7) and each was systematically coded to identify initial patterns in the data. Finally, codes were organized in broader categories that reflected different aspects of the data. The open-coding process was conducted by KÅ in discussion with other authors in an iterative process.

## 3 Results

**Fig. 1:**
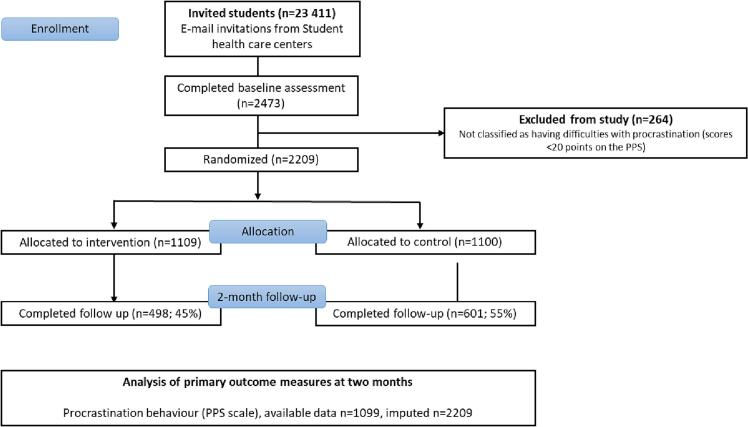
CONSORT flowchart.

Fig. 1 presents a participant flow diagram. From February 8 to 13, 2023, initial email invitations were sent to 23,411 students and two reminders sent one week apart to non-responders. A total of 2473 (11 %) students consented to participate and completed baseline assessment. There were 264 (11 %) consenters who were excluded due to not fulfilling the inclusion criteria. The remaining 2209 participants were randomized, 1109 to the intervention group and 1100 to the control group. Baseline characteristics of randomized participants are presented in Table 1. At follow-up, the primary outcome measure was collected from 50 % (*n* = 1099) of participants. Secondary measures (anxiety, stress, alcohol, sugary drinks, candy and snacks, physical activity) were collected from 49 % (*n* = 1077) of participants.

**Table 1:** Baseline characteristics of randomized participants.

	Total (*n* = 2209)	Intervention (*n* = 1109)	Control (*n* = 1100)
Sex, n (%)			
Women	1442 (65 %)	716 (65 %)	726 (66 %)
Men	749 (34 %)	383 (34 %)	366 (33 %)
Nonbinary or other	18 (1 %)	10 (1 %)	8 (1 %)
Age, mean years (SD)	26.4 (7.8)	26.5 (7.8)	26.2 (7.7)
Procrastination (PPS-score), mean (SD)	35.6 (9.4)	35.9 (9.4)	35.3 (9.4)
Decisional delay	30 %	30 %	30 %
Implemental delay	55 %	56 %	55 %
Timeliness and promptness delay	15 %	14 %	15 %

### 3.1 Primary and secondary outcomes

Descriptive statistics of primary and secondary outcomes are presented in Table 2 and estimates of effects of the intervention are presented in Table 3. Overall, findings were not different using available data versus imputed data. The evidence suggested no marked difference between groups regarding any of the outcomes, although there was weak evidence of lower physical activity in the intervention group.

**Table 2:** Descriptive statistics of primary and secondary outcomes.

	Intervention (*n* = 498)^a^	Control (*n* = 601)^a^
Primary outcomes (2-months)		
Procrastination behavior (PPS-score), mean (SD)	34.4 (9.5)	34.8 (9.9)

Secondary outcomes (2-months)		
Anxiety (GAD-7), mean (SD)	7.17 (5.34)	7.21 (5.44)
Stress (PSS-4), mean (SD)	6.46 (2.94)	6.49 (3.04)
Weekly alcohol consumption, mean (SD)	3.18 (4.46)	3.23 (5.30)
Heavy episodic drinking, mean (SD)	1.39 (1.89)	1.35 (1.91)
Sugary drinks (33 cl cans/week), mean (SD)	2.53 (3.64)	2.67 (3.68)
Candy and snacks (portions/week), mean (SD)	5.01 (5.66)	4.70 (5.24)
Moderate and vigorous physical activity (min/week), mean (SD)	308 (274)	334 (287)

a Based on primary outcome measures.

**Table 3:** Point estimates of effects on primary and secondary outcomes (intervention versus control).

	Available data analysis		Imputed data analysis	
	Est. 95 % CI	Probability of effect	Est. 95 % CI	Probability of effect
Primary outcomes (2-months)				
Procrastination (PPS-score)	−0.03 (−0.095; 0.033)	82.3 %	−0.034 (−0.095; 0.028)	85.9 %

Secondary outcomes (2-months)				
Anxiety (GAD-7)	0.0 (−0.109; 0.106)	50.1 %	−0.007 (−0.113; 0.101)	55.2 %
Stress (PSS-4)	−0.001 (−0.112; 0.104)	51.0 %	−0.004 (−0.111; 0.104)	53.0 %
Weekly alcohol consumption	1.023 (0.846; 1.235)	59.4 %	1.033 (0.863; 1.237)	63.7 %
Heavy episodic drinking	1.082 (0.929; 1.279)	83.6 %	1.082 (0.921; 1.256)	83.7 %
Sugary drinks (33 cl cans/week)	0.97 (0.826; 1.134)	65.1 %	0.961 (0.817; 1.127)	68.5 %
Candy and snacks (portions/week)	0.046 (−0.074; 0.161)	76.9 %	0.037 (−0.08; 0.15)	73.6 %
Moderate and vigorous physical activity (min/week)	−0.094 (−0.216; 0.022)	94.0 %	−0.1 (−0.217; 0.021)	94.9 %

Abbreviations:

Est. - Median of the marginal posterior distribution of adjusted standardized effects

CI - Compatibility interval (defined by the 2.5 % and 97 % percentiles of the posterior distribution).

Probability of effect – Proportion of the posterior probability greater/less than the null in the direction of the median of the marginal posterior distribution.

### 3.2 Ancillary analyses

**Fig. 2:**
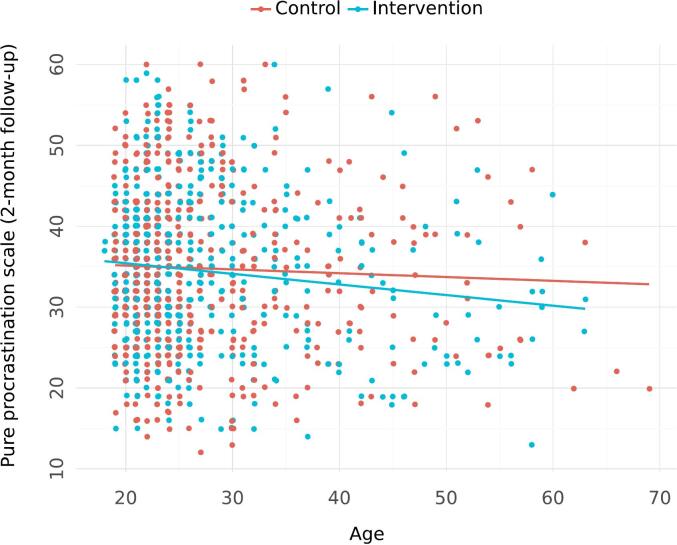
Effect modification with respect to pure procrastination scale at 2-month follow-up and baseline age (standardized interaction effect of −0.006 (95 % CI = −0.014; 0.001, probability of effect = 95.2 %)).

Effect modification analyses suggested that the intervention was more effective among older participants, with a group and age interaction standardized effect of −0.006 (95 % CI = −0.014; 0.001, probability of effect = 95.2 %, WAIC score = 1729.3 compared to 1729.9 for the non-interaction model). This attenuation of effect is depicted in Fig. 2, where one can see that the two lines representing control and intervention diverge as age increases.

**Fig. 3:**
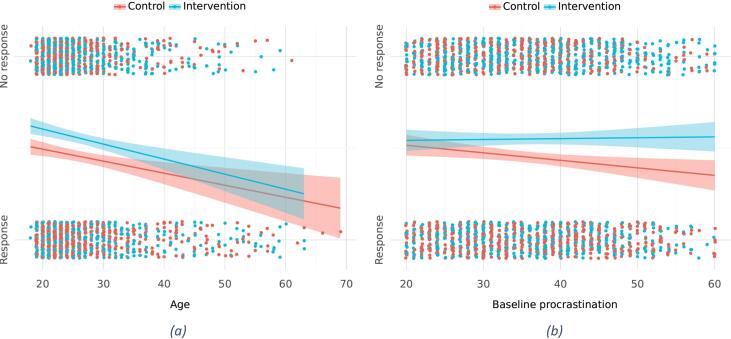
Response and no-response plotted against (a) age and (b) procrastination scores at baseline, stratified by randomized groups.

Attrition analyses indicated that participants in the intervention group were less likely to have reported a response at follow-up, as were younger participants irrespective of group. The difference between attrition in groups was explained by participants in the control group with higher PPS-score at baseline being more likely to respond to follow-up, compared to their counterparts in the intervention group. Fig. 3a and b depict these relationships, where it can be seen that older participants tended towards being responders (Fig. 3a) and that control group participants with higher baseline procrastination tended towards being responders to a greater degree than those in the intervention group (Fig. 3b).

### 3.3 User evaluation

Responses to the user evaluation questions are presented in Table 4. The findings indicate that 46 % of responding intervention group participants did not agree on having received support, scored a mean of 2.44 (on a 5-point Likert scale) on how well the intervention suited their needs, and scored a mean of 3.01 on whether they believed the support would be helpful for others.

**Table 4:** Descriptive statistics for perceived usefulness and acceptability of intervention.

	Mean (SD)	n (%)
Agree on having received support (*n* = 485)		Yes: 44 (9 %) No: 224 (46 %) Don't know: 217 (45 %)
How well intervention suited needs (*n* = 479) 1 = Not suited at all to 5 = Suited very well	2.44 (1.04)	1: 129 (27 %) 2: 66 (14 %) 3: 246 (51 %) 4: 21 (4 %) 5: 17 (4 %)
Believed support would be helpful for others (*n* = 433) 1 = Not very helpful to 5 = Very helpful	3.01 (1.04)	1: 50 (12 %) 2: 43 (10 %) 3: 231 (53 %) 4: 72 (17 %) 5: 37 (8 %)

In Table 5, main- and subcategories from open-ended responses are presented. In total, 452 comments from 452 individuals were collected of which 205 consisted of wordings such as “*no comment”*, or of random characters. In total, we analyzed 247 comments from which six main categories were developed: *understanding my challenges; resources and strategies; Focus did not support me; my need of support; no need of support; and do not recall receiving support.* Overall, findings uncovered a variety of views on procrastination and perceived problems that may follow. A majority of responses (*n* = 128) expressed that they felt no need for procrastination support and had not received, wanted, or sought any support. Among these, a considerable number of students (*n* = 81) explicitly stated that they had not received any support, suggesting that they did not perceive the intervention as supportive. Others stated that their procrastination difficulties were caused by, but also managed by, themselves and the circumstances of their everyday lives. Support or resources expressed as essential to deal with procrastination included having a good study technique and having access to social or psychological support. Those not feeling supported by Focus explained having troubles converting the advice they were given and implementing their intentions into action without continuous support.

**Table 5:** Main- and subcategories from open-ended responses explaining students´ needs and how Focus did or did not address them.

Main categories	Subcategories
Understanding my challenges with procrastination (*n* = 16)	Having many commitments creates stress (*n* = 8)
	My mental health creates challenges in my everyday life (*n* = 4)
	I am not sure if there is support available (n = 4)
Resources and strategies I use to cope with procrastination (*n* = 15)	Study technique – I remove distractions, make plans and set goals (n = 6)
	Social support – I receive support from people in my network (*n* = 5)
	Psychological support – I have previously, or am currently receiving therapy (n = 4)
Focus did not support me (*n* = 58)	The advice was useful but I have not managed to apply them or act differently (*n* = 27)
	The content was informative but after reading I immediately forgot about it (*n* = 20)
	Answering questions made me reflect on my habits (*n* = 7)
	I need to procrastinate to deliver my tasks (n = 4)
I need this kind of support (*n* = 30)	I want help with study technique, planning, and structure (n = 10)
	I want psychological support from a professional that I can talk to (*n* = 9)
	I need more hands-on support while making changes (n = 8)
	I do not know what kind of support I need, I only know my challenges (n = 3)
I do not procrastinate and do not need any support (*n* = 37)	I have no need, and therefore I have not looked for support (*n* = 37)
I do not recall receiving any support (*n* = 91)	I have not received any support (n = 81)
	I do not remember whether I have received support or not (n = 10)

## 4 Discussion

In this study we aimed to estimate the effects of a single session digital intervention to help university students in Sweden decrease their procrastination behaviors. Our results indicate that access to the single session intervention did not produce lower self-reported procrastination behaviors. Effect modification analyses did, however, indicate that older participants were better supported from the intervention, suggesting a possible masking of effects due to heterogeneity. Among secondary outcomes, there was evidence of a negative effect on physical activity. A narrative could be formed regarding the competing nature of day-to-day activities, i.e., if Focus led to more time spent on studies, then less time would be available for physical activity. However, this is speculative considering that the point estimate of effect on physical activity was small and the outcome was one of several secondary outcomes.

In contrast to CBT-treatment programs, which have a proven effect on procrastination among help seekers (Rozental et al., 2015a; Rozental et al., 2018; Eckert et al., 2018; Gagnon et al., 2019; Lukas and Berking, 2018; Mutter et al., 2023), the Focus intervention was designed to be proactive and to reach students not actively seeking support with their procrastination behaviors. Our user evaluation findings revealed that most students felt no need for procrastination support and did not experience receiving, wanting, or seeking any support because procrastination was not a problem for them. This positive student narrative on procrastination is also reflected in a qualitative study by Abramowski et al. (Abramowski, 2018), where procrastination was associated with further thinking time and attention to detail. Meanwhile, the individuals in our study who expressed a need for support highlighted that they had troubles converting the advice given through Focus and implementing their intentions into action without continuous support.

The Focus intervention was created in collaboration with Student health care centers across Sweden based on their request for a tool for procrastination that can reach students at scale. Additionally, previous research investigating health-related behaviors among university students has identified study-related stress and procrastination as barriers to not engage in healthy behaviors such as being physically active (Åsberg et al., 2022). While Focus was found to be insufficient in supporting students to change their procrastination behavior, there is still a need for future research to find avenues for a more preventive and proactive approach to procrastination and its negative consequences. This includes finding ways to reach students who experience mild to moderate difficulties but do not take action to seek support. A single session intervention as the one studied here could be accompanied by more comprehensive support to those students who may benefit. Recent insights from a consensus development panel within the field of digital mental health suggests that integrating digital interventions with face-to-face or virtual care can maximize their potential (Smith et al., 2023). However, the scalability of human guidance, such as at student health care centers, may not always be feasible due to a lack of resources, and it might not consistently align with individuals' needs and preferences for support. Moreover, social norms and stigma might deter individuals from seeking mental health support, creating an opportunity for further enhancing various forms of digital interventions (Carlbring et al., 2023). Treatment-seeking intentions could also increase through the use of a proactive single session intervention, as was the case in Ebert et al. (Ebert et al., 2019) where a brief intervention embedded in a mental health survey used tailored psychoeducation and information about mental health resources to increase students´ intentions to seek help.

### 4.1 Generalizability

We designed the recruitment procedures of this study to mimic how a possible real-world implementation of the intervention could be done, and participation was not overly burdensome. The inclusion criteria were broad, and we reached students by communication channels that they use in their daily lives and their contact details were obtained from a database including all students at each university. Therefore, we suggest that our findings should be interpreted as estimations of the effectiveness of the intervention. However, being a student in Sweden is not necessarily comparable to student life in other parts of the world, and so generalizability to other countries may therefore be limited. In addition, international students could not participate as Swedish was a requirement for participation.

In this study we wanted to exclude individuals who scored very low on the procrastination measure at baseline since they would not be the target group in a future intervention implementation. However, we did want to be inclusive, and therefore set the cut-off at 20 points (note that the range of PPS is 12–60), excluding only those who reported none or very little procrastination. We argue that this further strengthens the generalizability of our findings since we were not overly restrictive in who could participate in the study.

### 4.2 Limitations

The current study has a number of limitations that needs to be considered when interpreting the results. Firstly, the design and content of Focus differs from most psychological interventions for procrastination since there was no offer of therapeutic treatment. Instead, we designed Focus as a single session screening and advice intervention, with materials aiming to enhance self-reflection and stimulation of self-reinforcement (Kanfer and Gaelick-Buys, 1991).

The design of the trial necessitated that self-assessment of procrastination behaviors among all participants be done prior to randomization (all participants completed the PPS questionnaire at baseline). However, screening is also intended to be part of the Focus intervention, as it has been shown that self-assessment of behaviors may have an effect on later self-reported behavior (McCambridge et al., 2013; Bendtsen and McCambridge, 2021).

While we intended to compare Focus to a no treatment control condition, the study procedures may have instead led to a minimal active control condition (Goldberg et al., 2023). Thus, there is risk of bias towards the null due to the control group having effectively been given access to part of the intervention. Estimated effects may therefore be viewed as caused by the feedback and advice component only, assuming that there is no interaction between self-assessment and the later intervention materials. In other words, the feedback and advice component may not have been sufficient in this study to change participants' procrastination behaviors. This leads us to question if the minimal effect size that we did not want to miss when planning the trial (0.35 Cohen's d) was optimistic considering the brief nature of the intervention and the control condition being exposed to active components. Such an effect size could possibly be observed if the comparator was a group who were never contacted about procrastination, i.e., where no proactive intervention existed. With the current trial design, the implementation of Focus may have been justified if a smaller effect size of 0.1 Cohen's d was observed with a high probability of effect. However, the long-term outcomes from a population level effect on procrastination needs to be studied further in order to accurately decide on justifiable effect sizes.

Attrition bias is a concern in digital intervention research (Jakob et al., 2022). We anticipated a high attrition rate of 50 %, which was also the case. However, findings from analyses with imputed data were not different from available data analyses. Our sensitivity analyses showed a difference between responders and non-responders at follow-up based on their baseline PPS-score, with those reporting greater difficulties with procrastination being less likely to respond at follow-up in the intervention group but not so in the control group. In addition, younger individuals were less likely to respond to follow-up in both groups. A related limitation was the choice of a 2-month follow-up interval. This short follow-up was chosen so that participants would be more likely to remember their participation and to stay within the same academic semester, yet still enough removed from the intervention itself to decide if there were any lasting effects.

Finally, due to the very brief intervention requiring minimal participant contact, we did not include monitoring for deterioration effects. It is, however, important to acknowledge that research participants' conditions may worsen over the course of a study, and that monitoring of these trends is important to do when possible (Rozental et al., 2017). This consideration applies to both intervention and control group participants, with the latter potentially being negatively affected by the lack of treatment or the implicit assumption to wait for treatment, depending on the control condition design (Gunnarsson et al., 2023; Müssener et al., 2019).

## 5 Conclusions

Access to a single session of feedback and behavior change advice by means of a personalized website did not produce differential self-reported procrastination among university students who took the opportunity to self-assess their behaviors. The findings are limited by assessment reactivity due to screening at baseline and attrition to follow-up. More research is needed to develop proactive and effective interventions that can reach students at scale.

## Funding

This study was conducted under the auspices of the Swedish Research Council for Health, Working Life, and Welfare (Grant number 2018-01410; PI: ML). The funders had no role in study design, data collection and analysis, decision to publish, or preparation of the manuscript.

## Ethical approval

The study was approved by the Swedish Ethical Review Authority on 2022-08-24 (dnr 2022-00353). All participants gave informed consent after having been given full information about the study and its procedures. All procedures performed in studies involving human participants were in accordance with the ethical standards of the Swedish Research Council and with the 1964 Helsinki declaration and its later amendments or comparable ethical standards.

## Informed consent

Informed consent was obtained from all individual participants included in the study.

## Trial registration number

The trial was prospectively registered on 2023-02-03 (ISRCTN13533793).

## Declaration of competing interest

The author MB of this manuscript declare the following conflict of interest: I own a private company (Alexit AB) that develops and distributes eHealth solutions to the public and private sectors. Alexit AB played no role in developing the intervention, study design, data analysis, data interpretation, or writing of this report. Authors KÅ and ML declarations of interest: none.

## Appendix A Supplementary data

Appendix A Questionnaires. Appendix A
